# Somatostatin Analogue Treatment Primarily Induce miRNA Expression Changes and Up-Regulates Growth Inhibitory miR-7 and miR-148a in Neuroendocrine Cells

**DOI:** 10.3390/genes9070337

**Published:** 2018-07-04

**Authors:** Kristina B. V. Døssing, Christina Kjær, Jonas Vikeså, Tina Binderup, Ulrich Knigge, Michael D. Culler, Andreas Kjær, Birgitte Federspiel, Lennart Friis-Hansen

**Affiliations:** 1 Center for Genomic Medicine, Rigshospitalet, Blegdamsvej 9, 2100 Copenhagen, Denmark; kbdoessing@sund.ku.dk (K.B.V.D.); jonas.vikesaa@roche.com (J.V.); 2 Department of Clinical Physiology, Nuclear Medicine and PET, Rigshospitalet, Blegdamsvej 9, 2100 Copenhagen, Denmark; tina.binderup@rh.regionh.dk (T.B.); andreas.kjaer@rh.regionh.dk (A.K.); 3 Cluster for Molecular Imaging, Faculty of Health Sciences University of Copenhagen, Blegdamsvej 3B, 2100 Copenhagen, Denmark; 4 University College Copenhagen, Sigurdsgade 26, 2200 Copenhagen, Denmark; chkj@phmetropol.dk; 5 Department of Surgical Gastroenterology C, Rigshospitalet, University of Copenhagen, Blegdamsvej 9, 2100 Copenhagen, Denmark; ulrich.knigge@rh.regionh.dk; 6 Department of Clinical Endocrinology PE, Rigshospitalet, Blegdamsvej 9, 2100 Copenhagen, Denmark; 7 Biomeasure Incorporated/IPSEN, 650 W Kendall St, Cambridge, MA 02142, USA; mdculler@comcast.net; 8 Department of Pathology, Rigshospitalet, University of Copenhagen, Blegdamsvej 9, 2100 Copenhagen, Denmark; birgitte.federspiel@rh.regionh.dk; 9 Department of Clinical Biochemistry, Hillerød Hospital, Dyrhavevej 29, 3400 Hillerød, Demark

**Keywords:** somatostatin analogues, neuroendocrine tumors, cancer, miRNAs, miR-148a, miR-7, let-7

## Abstract

Somatostatin (SST) analogues are used to control the proliferation and symptoms of neuroendocrine tumors (NETs). MicroRNAs (miRNA) are small non-coding RNAs that modulate posttranscriptional gene expression. We wanted to characterize the miRNAs operating under the control of SST to elucidate to what extent they mediate STT actions. NCI-H727 carcinoid cell line was treated with either a chimeric SST/dopamine analogue; a SST or dopamine analogue for proliferation assays and for identifying differentially expressed miRNAs using miRNA microarray. The miRNAs induced by SST analogue treatment are investigated in carcinoid cell lines NCI-H727 and CNDT2 using in situ hybridization, qPCR and proliferation assays. SST analogues inhibited the growth of carcinoid cells more potently compared to the dopamine analogue. Principal Component Analysis (PCA) of the samples based on miRNA expression clearly separated the samples based on treatment. Two miRNAs which were highly induced by SST analogues, miR-7 and miR-148a, were shown to inhibit the proliferation of NCI-H727 and CNDT2 cells. SST analogues also produced a general up-regulation of the let-7 family members. SST analogues control and induce distinct miRNA expression patterns among which miR-7 and miR-148a both have growth inhibitory properties.

## 1. Introduction

Gastro-Entero-Pancreatic neuroendocrine neoplasms (GEP-NEN) are generally slow growing tumors originating from neuroendocrine cells in the gastro-intestinal tract, the diffuse neuroendocrine system, pancreas and the bronco-pulmonary system [1]. According to the World Health Organization (WHO) 2010 classification [2] GEP-NEN can be classified according to Ki67 index and/or mitotic count into neuroendocrine tumors (NETs, NET-G1 and NET-G2) and neuroendocrine carcinomas (NEC G3) [2,3]. The bronco-pulmonary NENs are classified according to mitoses pr. 2 mm^2^ and the presence or absence of necrosis according to the 2015 WHO classification [4]. This separates small-cell and large-cell, poorly differentiated based on mitosis only, and the typical carcinoids (TCs) and atypical carcinoids (ACs) by <2 mitoses per 2 mm^2^/no necrosis and 2–10 mitoses per 2 mm^2^/with focal necrosis. In general, NETs of the lung comprise <20% of all lung cancers, and TCs and ACs comprise 1–2% of these [4,5]. The yearly incidence of GEP-NENs and bronco-pulmonary NENs is 2–3 and 0.2–2 per 1,000,000 inhabitants respectively, but this has been increasing during the last decades [1,6,7,8]. TCs share homologies with G1 GEP-NENs and ACs with G2 GEP-NENS coinciding with the fact that ACs are more malignant in nature and more prone to metastasize compared to TCs [8,9]. The clinical picture depends on the site of the primary tumor and its ability to secrete neuroamines and/or peptides at supra-physiological levels (functioning tumors) as the clinical syndrome depends on the transmitter/hormone secreted [10]. However, most of the GEP-NENs and bronco-pulmonary NENs are silent or ‘non- functional’, as they either do not secrete any transmitters/hormones or the one they secrete does not cause clinical symptoms [10,11]. The lack of endocrine symptoms in these patients often delays the diagnosis until the presence of symptoms caused by the mass effect and/or the presence of metastases, mainly hepatic metastases [12]. In patients with localized low grade GEP-NETs, the 5- year survival rate is 60–100% whereas patients with regional disease or distant metastases have 40% and 29% 5-year survival rates respectively [13]. In bronco-pulmonary NENs the overall survival is higher in TCs versus ACs with a 5-year survival rate of 90% and 60% respectively and increases with resectable tumors [14]. 

A common feature for endocrine cells is that both their secretion and growth can be inhibited by somatostatin (SST) [15]. In the nervous tissue, SST acts like a neurotransmitter and a neuromodulator. In the gastrointestinal tract, SST is expressed by specialized endocrine cells and acts as a paracrine factor [16]. The somatostatin receptors (SSTRs) belong to the G-protein coupled receptor family and there are five different SSTR subtypes (SSTR1-5), all differently expressed by neuroendocrine cells [17]. Seventy to ninety percent of all GEP-NET express SSTRs and tumors expressing SSTRs often contain one or more receptor subtypes, most often SSTR1, 2, 3 and 5, whereas SSTR4 is less commonly seen in endocrine tumors [18]. This has made SST useful for both visualization and treatment [19,20]. The expression is also related to the tumor grade, since the receptors are preferably expressed in low grade tumors, whereas the expression of some receptor subtypes is reduced in the more dedifferentiated tumors [21]. The different SSTR subtypes all bind the endogenous ligands; SST14, SST 28 and cortistatin; with equally high affinity in the nanomolar (nM) range. The widespread expression of SSTRs by a large number of human tumors and the biological actions of SSTRs form the basis for in vivo tumor targeting. However, the short half-life in circulation (1–3 min) of the endogenous SST peptides makes them unsuited for therapy [22]. Therefore a number of synthetic analogues with longer half-lives, such as somatostatin SMS201-995 (Octreotide), RC-160 (Vapreotide), BIM-23014 (Lanreotide), MK678 (Seglitide) and SOM-230 (Pasireotide LAR), were developed and are currently in clinical use [23,24]. Surgical removal is the optimal treatment for GEP-NENs and bronco-pulmonary NENs, and somatostatin analogue (SSA) treatment constitutes the gold standard for symptomatic and anti-proliferative control [8,25].

MicroRNAs (miRNAs) are small non-coding RNA molecules (on average 20–23 nucleotide (nt) long) that modulate gene expression by binding to complementary sequences on target messenger RNA (mRNAs). This predominantly results in a decrease of target mRNA levels [26]. An increasing number of studies indicate that miRNAs are involved in many important biological processes, including proliferation, apoptosis, differentiation, angiogenesis and immune response. miRNA deregulation leads to aberrant gene expression in various diseases and dysregulation of miRNA expression have been shown to be involved in cancer development [27]. We therefore wanted to examine which miRNA operate under the control of SST and if they contribute to the growth- inhibitory effects of SST.

## 2. Materials and Methods 

### 2.1. Cell Lines and Tissue Culture

Four human carcinoid cell lines, two intestinal (CNDT2 and HC45) and two of pulmonary origin (NCI-H720 and NCI-H727) were used for experiments.

CNDT2 is a human midgut carcinoid cell line kindly provided by Lee M. Ellis M.D. Anderson Center Texas USA [28] and grown in DMEM/F12 with 15 mM HEPES (Life Technologies, Carlsbad, CA, USA) supplemented with 10% FBS (Th. Geyer GmbH, Stuttgart, Germany), penicillin 100 U/mL and streptomycin 100 µg/mL (Life Technologies), 5 mL Sodium pyruvate 100 mM (Sigma-Aldrich, St. Louis, MO, USA), 5 mL MEM NEAA 100x (Life Technologies), 5 mL L-Glutamine 200 mM 100x (Life) and 10 ng/mL NGF (Life Technologies) and kept at 37 °C/5% CO_2_. HC45 is a human ileal carcinoid cell line kindly provided by Ricardo V. Lloyd Mayo Clinic [29] and kept in RPMI 1640/Glutamax (GIBCO, Waltham, MA, USA) supplemented with 10% FBS, 1% P/S and 10 ng/mL Insulin (Invitrogen, Carlsbad, CA, USA) at 37 °C and 5% CO_2_.

NCI-H720 is an atypical and NCI-H727 is a typical human pulmonary carcinoid cell line obtained from ATCC (Boras, Sweden). Both cell lines were cultured in RPMI 1640 Glutamax supplemented with 10% FBS, penicillin 100 U/mL and streptomycin 100 µg/mL, 1 mM Sodium Pyruvate (Life Technologies) at 37 °C and 5% CO_2_.

For experiments involving seeding cells into new plates, cells were always allowed to adhere overnight.

### 2.2. Tumor Tissue

Five formalin-fixed and paraffin-embedded (FFPE) tissue samples of carcinoid tumors (see Table 1), obtained from the Department of Pathology (Rigshospitalet, Copenhagen, Denmark), were used for Laser Capture Microdissection, qPCR and in situ hybridization, see descriptions for the procedures below. 

### 2.3. Somatostatin Analogues

Two SST analogues (BIM-23014 (Lanreotide) and BIM-23023), a combined SST-dopamine 2 receptor analogue (BIM-23A760) and a pure dopamine receptor D2 (DRD2) analogue (BIM-53097) were used. The compounds’ binding affinities for the SST and dopamine receptors are shown in (Table 2). The compounds were initially dissolved in 100 µL 99% EtOH then in 0.1 N acetic acid/0.1% BSA to a final stock concentration of 10^−3^ M and used for experiments at suitable concentrations in complete growth medium.

### 2.4. Transfection Studies and Cell Growth Analyses

A total of 4 × 10^6^ NCI-H727 or 1.5 × 10^6^ CNDT2 cells were seeded and used for each transfection. To 965 µL Opti-MEM (Invitrogen) 10 µL Negative control 1 or 2 (Thermo Scientific, Waltham, MA, USA) or mature miRNA miR-7/miR-148a (Applied Biosystems, Carlsbad, CA, USA) or inhibitor—miR-7 LNA/miR- 148a LNA (Exiqon, Vedbæk, Denmark) was added to a final concentration of 50 nM together with 25 µL Turbofect transfection reagent (Fermentas, Leon-Rot, Germany) in 5 mL complete growth medium and left to incubate for 15–20 min at room temperature (RT) before being added drop-wise to the cells.

For growth analyses, 4 × 10^4^ cells (NCI-H727) or 1.5 × 10^4^ cells (CNDT2) were seeded in each well into E-plates for use in the xCELLigence system (Roche/ACEA, San Diego, CA, USA) for proliferation studies. The xCELLigence analyzer, which is an electronic cell sensor array technology, allows label-free and real-time monitoring of cell proliferation. The presence of the cells on top of the electrodes will affect the local ionic environment at the electrode/solution interface, leading to an increase in the electrode impedance. The more cells that are attached to the electrodes, the larger the increases in electrode impedance. For further details, see references [32,33]. The difference in cell number seeding for growth assays is due to differences in size and proliferative rate between the two cell lines. A series of analogue concentrations were used to find the optimal concentration for the actual growth experiments. The analogues were added daily directly to the wells of the E-plates in complete growth medium used for the normal passage of cells, without changing it for the duration of the entire growth experiment. The concentrations of the analogues for growth experiments were BIM-23a760 10^−7^ M, BIM-23023 10^−7^ M, BIM-23014 10^−9^ M and finally BIM-53097 10^−9^ M. 

### 2.5. RNA Extraction

Total RNA was extracted using Trizol reagent (Life Technologies) according to the manufactures specifications. The RNA concentration was measured on the NanoDrop (Thermo Fisher Scientific, Wilmington, DE, USA) and the integrity determined using the Agilent 2100 Bioanalyzer (Agilent Technologies, Santa Clara, CA, USA).

### 2.6. Somatostatin Receptors mRNA Quantification

The expressions of the SSTRs were quantitated as previously described [34] and normalized to the expression of β-actin.

### 2.7. Laser Capture Microdissection

Tumor and normal cells from FFPE tissue, see tissue specifications above, were Laser Capture Microdissected (LCM) using an Arcturus LCM system (ThermoFisher Scientific, Waltham, MA, USA). 10 µm sections of tissue were mounted on Pen Membrane slides (Applied Biosystems, Foster City, CA, USA). Sections were stained with cresyl violet, using the LCM staining kit (Ambion, Foster City, CA, USA/Life Technologies, Carlsbad, CA, USA) with cresyl violet according to the manufacturer’s instructions. After tissue sections had been collected and transferred to the collection cap, the cap was immediately transferred and clicked on to an Eppendorf tube containing the 100 µL of the Lysis Solution used in the first step of the RNAqueous^®^-Micro Kit (ThermoFisher Scientific). The tube was inverted to ensure complete coverage of the dissected cells in the buffer. RNA isolation was performed according to the manufacturer’s instructions.

### 2.8. qPCR of microRNA Expression

The expression of miR-7 and miR-148a was quantitated using TaqMan miRNA assay (Applied Biosystems). Briefly, 100 ng of the total RNA was reversely transcribed into cDNA using gene specific primers and the TaqMan MicroRNA Reverse Transcription Kit (Applied Biosystems) according to the manufacturer’s protocol. Samples were run on the ABI PRISM 7900 HT Sequence Detection System (Applied Biosystems). For normalization of the miRNA expression data the geometric mean of hsa-miR-191 and RNU-44 were used [35]. Primer sequences are listed in Table 3.

### 2.9. In Situ Hybridization

Formalin-fixed and paraffin-embedded (FFPE) tissue samples of carcinoid tumors were obtained from the Department of Pathology (Rigshospitalet, Copenhagen, Denmark). A double-DIG-labeled LNA-modified oligos miR-7 (Exiqon, Munich, Germany), probe sequence 5′-ACAACAAAATCACTAGTCTTCCA-3′, RNA-T*m* 80 °C and double-DIG-labeled LNA-modified oligos miR-148a (Exiqon), probe sequence 5′-ACAAAGTTCTGTAGTGCACTGA-3′, RNA-Tm 80 °C were used for detection as described [36]. Probe concentration was 100 nM and slides were hybridized at 50 °C. Sections were counterstained with Nuclear Fast Red. Pictures of representative areas of the slides were taken with a Zeiss Axio Imager (Zeiss, Jena, Germany), original magnification × 20/10. Cells with intense blue nuclear stain were scored as positive. The level of expression within a positive cell was not scored. A LNA probe against snRNA U6 (Exiqon) was used as positive control and a scramble probe (Exiqon) as negative control.

### 2.10. MicroRNA Microarray

For microarray analysis 1 µg of total RNA was labeled using the Flashtag RNA labeling kit for Affymetrix (Genisphere LLC., Hatfield, PA, USA) according to the manufacturer’s instructions. The labeled samples were hybridized to GeneChip miRNA Array (Affymetrix, Santa Clara, CA, USA). The Affymetrix miRNA array assay miRNAs includes small nucleolar RNAs (snoRNAs) and small Cajal Body specific RNAs (scaRNAs) in human. The 847 human miRNAs on the array are derived from Sanger miRbase miRNA database V11. Four copies of each miRNA probe are distributed on the array.

### 2.11. RNA Profiling

Arrays were washed and stained with phycoerytrin conjugated streptavidin (SAPE) using the Affymetrix Fluidics Station^®^ 450, and the arrays were scanned in the Affymetrix GeneArray^®^ 3000 scanner to generate fluorescent images, as described in the Affymetrix Gene Chip^®^ protocol. To minimize batch variation, equal numbers of treatment groups were included in each batch.

### 2.12. Data Analysis

Raw data files were imported into Affymetrix’s miRNA QC Tool (Affymetrix) and normalized using the quantiles normalization and median Polish summarization following a background correction that corrects for the GC content of the each particular probe. Log2 intensities of the 847 human miRNAs were imported into the Data Analysis software package Qlucore Omnics Explorer v2.1. Principal Component Analysis (PCA) visualization of the clustering of samples using the genes selected in the class comparison was performed using the build-in PCA tool in Qlucore Omnics Explorer v2.1 (Qlucore AB, Lund, Sweden).

Class comparison analysis was performed using Students *t*-test. A multiple comparison test was used for all three tissue-types and a two-group comparison was used for comparing AP versus NF. The miRNA was defined as being differentially expressed between the compared groups if the *p* value was less than 0.05 and the fold change above 1.5.

### 2.13. Statistical Analyses

Students’ unpaired *t*-test was used and differences with a *p* ≤ 0.05 were considered significant and indicated by *. Unless otherwise stated results are given as median ± standard deviation (SD). 

## 3. Results

### 3.1. Somatostatin and Dopamine Analogues Inhibit the Growth of a Carcinoid Cell Line NCI-H727

**Figure 1:**
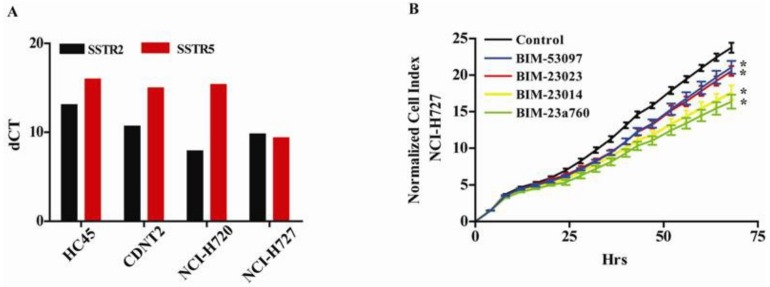
Carcinoid cell lines express all somatostatin receptors (SSRTs), and activation of both SSTRs and DRD2 gives the most potent growth inhibition of a carcinoid cell line (**A**) All the four examined carcinoid cell lines express SSTR subtype 2 and 5 when analyzed by qPCR (**B**) The growth of NCI-H727 is inhibited by somatostatin, dopamine and the chimeric somatostatin-dopamine agonists. The dopamine analogue BIM-53097 (blue) and the somatostatin analogues (SSAs) BIM—23023 (red) were the weaker inhibitors of cell growth. The somatostatin agonist BIM-23014 (yellow) and the chimeric somatostatin-dopamine compound BIM-23A760 (green) were the stronger inhibitors of carcinoid cell growth. The control (black) is vehicle without agonist.

We first examined the expression of the SSTRs in four different carcinoid cell lines, HC45 and CNDT2 (both intestinal) and the atypical NCI-H720 and typical NCI-H727 pulmonary cell lines, in order to choose an optimal cell line as a model system for examining the effect of SSAs on miRNA expression in NETs. All the carcinoid cell lines expressed SSTR subtypes 2 and 5 Figure 1A, and for further analyses we selected two of the cell lines with highest SSTR2 mRNA level and each representing common NET origins in lung (NCI-H727) and intestinal (CNDT2). Furthermore, the HC45 proved very difficult to grow even after having been immortalized by retroviral transfection with a constitutive active human Telomerase Reverse Transcriptase TERT expression vector and we discontinued using this cell line. We have also previously shown that the CNDT2 and NCI-H727 cell lines are good model systems when examining NETs [37] and proceed with these two cell lines as our model. 

Having shown that NCI-H727 cells expressed SSTR2- and five subtypes we examined the effect of the SST and dopamine analogues on their growth. We found that all compounds inhibited the growth of the carcinoid cell line (Figure 1B). The most potent inhibitor of proliferation was the combined SSTR and dopamine receptor DR agonist BIM-23A760 (green) followed by the SSTR agonist BIM-23014 (yellow) and BIM-23023 (red). The least effective of the SSAs was the DR agonist BIM-53097 (blue) Figure 1B.

### 3.2. Somatostatin Receptor Activation Primarily Induces microRNA Expression Changes

**Figure 2:**
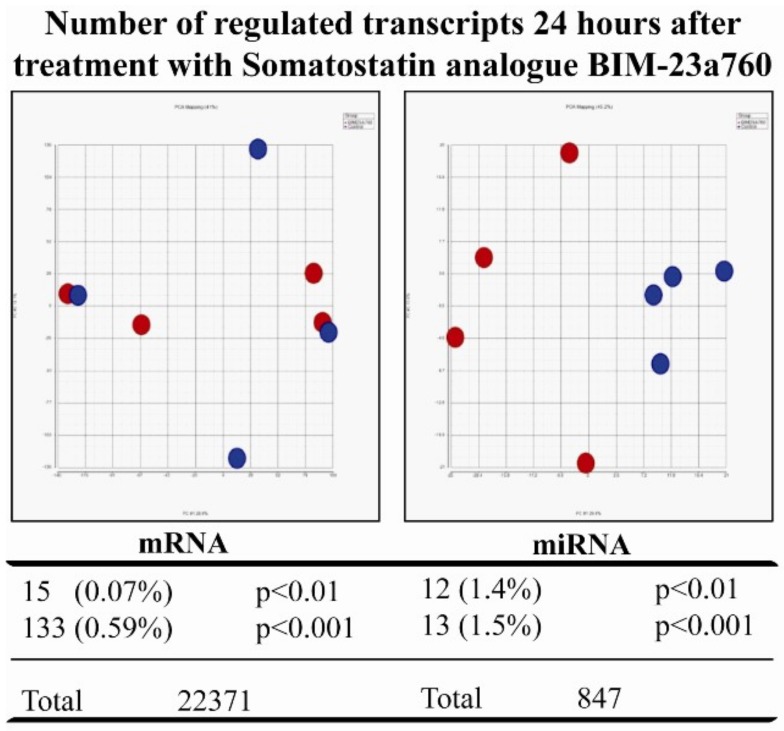
The dual somatostatin-dopamine agonist BIM-23a760 induces greater changes in microRNA (miRNA) than in messenger RNA (mRNA). Carcinoid NCI-H727 cells were treated with the dual somatostatin-dopamine agonist BIM-23a760 for 24 h and the changes in mRNA and miRNA were examined using microarray. Principal component analysis (PCA) of the changes in mRNA and miRNA demonstrated that mRNA based PCA did not clearly separate the BIM-23a760 treated cells (shown in red) from the control cells treated with vehicle without BIM-23a76 (shown in blue). In contrast a PCA based on the changes in miRNA expression clearly separated the treated cells (shown in red) from the untreated cells (shown in blue). This suggests that the miRNA changes induced by BIM-23a760 are more specific than the changes in mRNA expression. This was also supported by the fact, that a higher proportion of miRNA transcripts than of mRNA are changed by BIM-23a760 treatment.

After having seen that SST analogues inhibit growth we treated NCI-H727 cells with the most potent inhibitor—BIM-23A760—and compared the mRNA and miRNA profiles of treated cells with that of untreated cells (Figure 2). PCA plots based on either mRNAs or miRNAs of cells treated with SST analogue showed that PCA based miRNA expression profiles clearly separated controls from treated cells. In contrast, PCA based mRNA profiles did not (Figure 2). Furthermore, when calculating the number of transcripts changed and regulated by SST 24 h after treatment it is clear that treating with SST regulates miRNAs to a higher degree than mRNAs (Figure 2).

### 3.3. Somatostatin Induces Distinct Receptor Based/Activated microRNA Expression Profiles and Particularly Up-Regulates miR-7 and miR-148a

**Figure 3:**
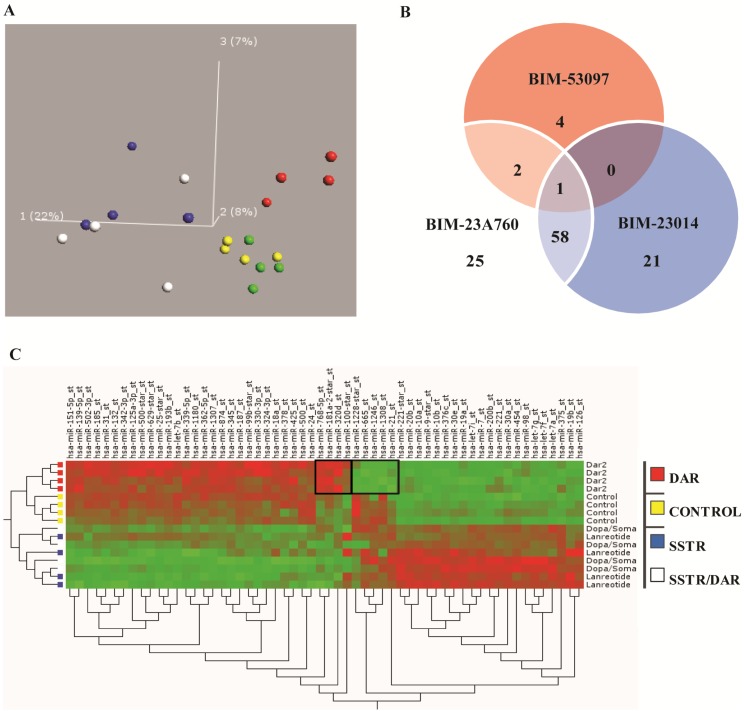
PCA shows specific miRNA expression depending on receptor activation. (**A**) The unbiased PCA separates the samples based on variation in the expression of miRNA in each sample. In the first dimension samples treated with BIM-23014 (blue—SSTR agonist) and BIM- 23A760 (white—SSTR and dopamine receptor D2 (DRD2) chimeric agonist) separated from the others. BIM-23023 (green—SSTR) did not separate from the control (yellow). Also in the first dimension BIM-53097 (red—DRD2) did not separate from the controls. However, in the third dimension the BIM-53097 (red—DRD2) treated samples clearly separated from the controls. The percentage indicates how much of the total variation is present in each dimension. In this experiment 22% of the total variation of miRNA expression is found in the first dimension, 8% in the second and 7% in the third. The two most potent inhibitors of growth BIM-23A760 and BIM-23014 group together, the DRD2 agonist and the least effective growth inhibitor separates by itself. (**B**) The Venn diagram shows the miRNAs shared between the different analogue treatments and that the number of miRNAs shared between BIM-23A760 (SSTR-DRD2) and BIM-23014 (SSTR) are greater than BIM-53097 (DRD2). (**C**) On the right side of the map is the clustering of treatment where DRD2 and controls separate from chimeric compound BIM-23A760 (SSTR-DRD2) and BIM-23014 (SSTR). Furthermore it seems to separate controls and DRD2. Notice in the middle of the control/DRD2 treatment, there is a small fraction of miRNA which seems to be controlled by dopamine alone and different from the control group.

Having demonstrated that SST and dopamine analogues inhibited the growth of the carcinoid cell line NCI-H727 and that activation of these receptors primarily affected miRNA expression, we hypothesized that at least some of their growth inhibitory effects depended on receptor activation and miRNA expression regulation. Also, after identifying the most potent inhibitor of growth among the different SST analogues we expanded the study and examined all the different SST analogues’ effect on miRNA expression by miRNA analysis of NCI-H727 cells treated with BIM-23A760, BIM-23014, BIM-23023, BIM-53097 and with a control. A PCA of the miRNA expression profiles showed that, depending on which analogue the cells were treated with, they could be separated into groups according to the analogue used and receptor(s) activated. In the first dimension the samples treated with BIM-23a760 (SSTR/DRD2) and BIM-23014 (SSTR-only) analogues separated from the rest and in the third dimension there was a difference in miRNA expression depending on receptor type activation (Figure 3A).

A Venn diagram shows the overlap between miRNA expression shared between the analogues and analogue treatment. The results show that a higher number of miRNAs are affected and changed after treatments with BIM-23a760 and BIM-23014, which target SSTR either alone or in combination with DRD2, compared to BIM-52097 which only targets DRD2. The highest degree of miRNA expressional changes was induced by SSTR activation compared to selective DRD2 activation (Figure 3B). However, a more specific miRNA expression change was observed in the small fraction of miRNAs changed only in response to BIM-53097 and DRD2 receptor activation. Since BIM-23023 (SSTR) did not separate from the control group in the PCA plot, it was excluded (Figure 3B). Based on the miRNA array results we created a heat map to better visualize the changes in miRNA expression between the treatments with the different compounds, again data with BIM-23023 (SSTR) being excluded (Figure 3C). We also created a list of the most significantly down- or up regulated miRNAs from the two compounds which showed the biggest inhibitory effect on cell proliferation BIM-23A760 (SSTR/DRD2), BIM-23014 (SSTR) and the least effective growth inhibitor BIM-53097 (DRD2) (Table 4). From this list we chose to focus on miR-7 and miR-148a.

### 3.4. In Situ Hybridization and qPCR on Neuroendocrine Tumors and Neuroendocrine Tumor Laser Capture Microdissected Tissue Confirms the Presence of miR-7 and miR-148a 

**Figure 4:**
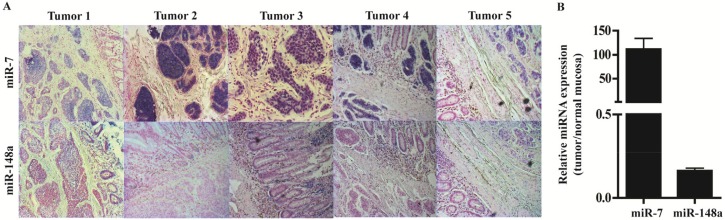
miR-7 and miR-148a are primarily expressed in neuroendocrine cells and inhibit the growth of a carcinoid cell line. (**A**) In situ hybridization of miR-7 show robust expression of miR-7 specifically located to the neuroendocrine cells. The expression of miR-148a is also predominantly seen in the neuroendocrine cells, although to a lesser extent than miR-7. (**B**) qPCR of laser capture micro dissected cells confirmed that miR-7 is robustly expressed in NETs compared to normal mucosa. In contrast, the expression of miR-148a is lower in NETs than in the normal mucosa that contains endocrine cells.

To characterize the localization and to visualize the expression of miR-7 and miR-148a in NETs we performed in situ hybridization on five NETs and found miR-7 expressed exclusively by the endocrine cells, both tumor and normal cells; miR-148a was also expressed primarily by the endocrine cells also both tumor and normal cells but to a lesser extent (Figure 4A). Expression analysis of LCM NET tissue confirmed the up-regulation of particularly miR-7, but also shows the presence of miR-148a (Figure 4B).

### 3.5. miR-7 and miR-148a Modulate the Growth of NCI-H727 and CNDT2 Carcinoid Cell Lines 

**Figure 5:**
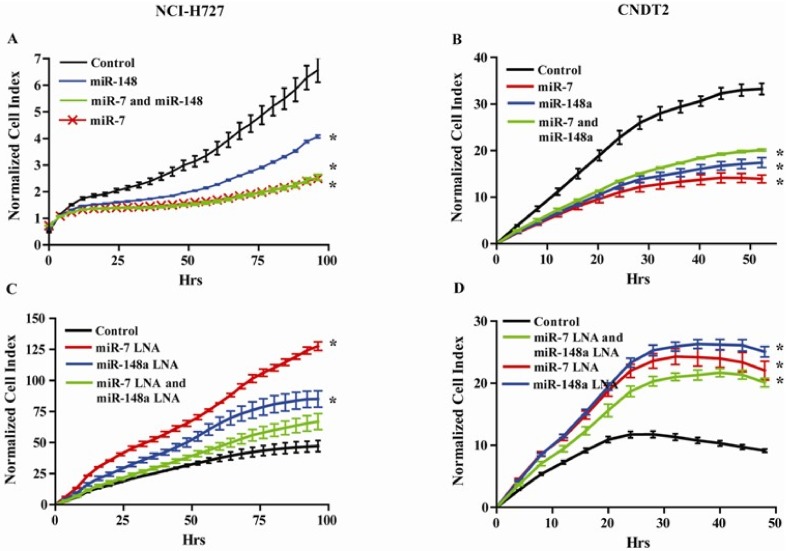
miR-7 and miR-148a regulates growth of the carcinoid cell lines NCI-H727 and CNDT2. (**A**) and (**B**) Both miR-7 and miR-148a reduces cellular growth of the carcinoid cell lines NCI-H727 and CNDT2 where the highest inhibitory effect is caused by transfecting with miR-7. (**C**) and (**D**) Inhibiting miR-7 and miR-148a by transfecting the carcinoid cell lines NCI-H727 and CNDT2 with miRNA inhibitors alleviate growth repression and cause the cells to grow more than the controls. * growth rates statistically significant compared to control.

Having demonstrated that SST analogues induced the expression of miR-7 and miR-148a and that both these miRNAs are expressed in NETs, we examined how these miRNAs would affect the growth of both the NCI-H727 and CNDT2 carcinoid cell lines by either over expressing or inhibiting them. Both miR-7 and miR-148a significantly inhibit the growth of carcinoid cells in vitro indicating that these miRNA could mediate some of the growth inhibitory effect induced by SST analogues (Figure 5A,B). We subsequently blocked the effect of miR-7 and miR-148a by transfecting LNA-inhibitors that increased the growth of both carcinoid cell lines (Figure 5C,D). 

### 3.6. Somatostatin Modulates the Expression of the Let-7 Family

We have previously shown that the expression of several Let-7 family members is reduced during NET carcinogenesis [37] and we therefore specifically examined how the SSTR and DRD2 analogues affected the expression of the let-7 miRNA family. We found that the expression of 4 of the Let-7 members increased after treatment with SSTR analogues, the expression of one was slightly reduced and the expression of 4 was unaffected (Table 5). 

Thus, SST analogues had the ability to reintroduce the expression of the let-7 family and possibly revert some of the pathways otherwise involved in NET carcinogenesis and malignancy.

## 4. Discussion

We have shown that in our NET model system SST analogue treatment primarily induces changes in miRNA expression profiles in carcinoid cell lines. SST analogues are widely used to treat patients with NETs, as the SST analogues both alleviate the carcinoid syndrome and inhibit growth of the tumors [19,38,39,40]. Here we show that SST analogues inhibit the growth of two carcinoid cell lines and that the inhibitory potential depends on the analogue used. This is in good concordance with other studies that also show that SST analogues inhibit the growth of the NCI-H727 carcinoid cell line [30,41] and the CNDT2 carcinoid cell line [42] and that a chimeric compound targeting SSTRs and DRD2s has the most potent growth inhibitory effect [30,41,42]. The growth inhibitory effects of SST analogues are mediated directly by binding of SST to the SSTRs, which leads to cell cycle arrest or apoptosis and indirectly by the inhibition of growth factors and the suppression of oncogenic signal transducing pathways. However, the molecular mechanisms linking neuroendocrine proliferation and tumor progression are not yet fully understood. The PI3K/MAPK/mTOR pathway is known to play an important role in NETs [43,44]. Already existing therapies used to treat patients with NETs consist of agents targeting this specific signaling pathway [45,46,47,48,49]. SSTR2 has been shown to bind directly to the p85 subunit of PI3K, which belongs to the class I_A_ PI3Ks and is the class specifically involved in promoting cell survival, growth and proliferation and the most important subclass involved in human cancers [50,51]. SST analogue treatment of a pancreatic cell line can inhibit the binding between SSTR2 and p85 which is critical for the down-regulation of PI3K activity and resulting decreased cell survival [50].

We found miR-7 and miR-148a among the most up-regulated miRNAs after treatment with SST analogues and increased expression of the let-7 family members, all of which have growth inhibitory effects. miR-7 has been shown to be down-regulated in several cancers including colorectal cancer [52] and gastric cancer [53] and to be endocrine specific [54], with evidence going both ways to whether or not miR-7 acts as a tumor suppressor [55]. Here we demonstrated miR-7 to be highly present in NETs. Both miR-7 and SST targets the PI3K/MAPK/mTOR pathway underlining the significance for the up-regulation of this specific miRNA by SST. In a study in lung cancers, including the carcinoid cell line NCI-H727 showed miR-7 to directly target PI3KR3, the regulatory subunit of PI3K, and to reduce the metastatic potential by reducing the effect of TLR9 signaling [56]. In hepatocellular carcinoma miR-7 has been shown to directly target and repress PI3KCD an integral component of the PI3K signaling pathway, thereby both inhibiting cellular growth, invasion and migration in vitro and more importantly tumorigenesis and metastasis in vivo [57].

Another important aspect of the induction of miR-7 expression in cancer lies in the fact that miR-7, through its inhibitory actions on central cancerous signaling pathways, can increase the sensitivity and improve otherwise chemo-or radiotherapy resistant tumor cells [55]. SST mediates up- regulation of miR-7 thereby increasing the cells sensitivity to the growth inhibitory actions of SST itself resulting in a positive feed-forward loop.

miR-148a has been shown to be down-regulated in early gastric- [58], pancreatic- [59] and colon cancer [60], as well as in breast cancer [61], and, when down-regulated, suggested as a biomarker for the diagnosis and prognosis of gastrointestinal cancers [58,62,63]. Shivapurkar et al. showed that a panel of six miRNAs including miR-148a could predict the risk of recurrence of colon cancer [60]; thus the up-regulation of miR-148a by SST analogue treatment might also be used as a biomarker in NETs. We found miR-148a localized to endocrine cells and tumor tissue, all of which points towards it being a specific and important miRNA in endocrine tumor formation and initiation. Our finding that miR-148a has a growth inhibitory effect on carcinoid cell lines makes it an important miRNA to up-regulate for SST analogues as it will give the SST analogues the ability to “pack that extra punch” in stopping cell growth, and which is possibly why we see that SST analogue treatment primarily gives a change in miRNA expression rather than mRNA expression.

SST itself attenuates Insulin Growth Factor 1 (IGF-1) signaling [64] and IGF-1 Receptor (IGF-1R) is important in GEP-NET tumor growth factor biology [51]. Since miR-148a targets IGF-1, SST analogue induced miR-148a expression could again lead to the interference with the PI3K signaling pathway by the inhibition of growth factors like IGF-1 and its receptor IGF-1R, which frequently are overexpressed in NETs [43,51]. Inhibition of IGF-1R signaling has been shown to decrease PI3K signaling and the induction of cell cycle arrest and apoptosis [51]. miR-148a directly targets and down-regulates IGF-1R in breast cancer and over-expression of miR-148a decreased phosphorylated Akt, a component of the PI3K signaling pathway [61]. miR-7 also targets IGF-1R in a gastric cancer model with significant influence on inhibiting the metastatic potential in this model [53]. However, while SSTR agonists have been shown to have anti-tumor growth activity [30,41,42], IGF-1R inhibition proved to be ineffective in treatment of NETs [65]. One explanation could be that IGF-1R inhibition only targets the receptor activity but not directly the downstream signaling events. In contrast, SSTR activation and induction of the miRNAs could potentially inhibit IGF-1R signaling at multiple levels and hence be more effective. We have previously shown the let-7 family to be among the most down-regulated miRNA in NETs. We also identified and that it targets HMGA2, BACH1 and MMP1 and reduce the expression of these oncogenes all present in NETs [37]. Here we find that treatment with SST analogues modulates the expression of the let-7 family and leads to the up-regulation of several family members and the down-regulation of only one.

Recently, several reports have established the let-7 miRNAs as key players in metabolic pathways, especially in the glucose metabolic pathway through the inhibition of IGF-1R, which is a key target in the PI3K/mTOR signaling pathway [66]. Others have shown the let-7 family to directly target and inhibit IGF-1 and IGF-1R, which harbors three let-7 binding sites in its 3’UTR. This leads to the elimination of PI3K activation, and hence abrogates the signaling pathway leading towards cell division, differentiation and survival. placing let-7 up-stream of IGF-1/IGF-1R with downstream effects on the PI3K signaling cascade [67,68]. The positive modulation of the let-7 family by SST analogues targets and inhibits the IGF-1/PI3K signaling pathway in addition to maybe inhibiting glucose nutrient in aiding the rapid growth of the cancer cells. By this mechanism, they are targeted on both survival signaling as well as their supply of nutrients. In conclusion miR-7, miR-148a and the let-7 family most up-regulated by SST have been shown to play a role in inhibiting the PI3K signaling pathway at different levels reducing the cancer cell’s ability to escape and circumvent inhibition of a single step. The fact that the miRNAs up-regulated by the SST analogues, the analogues themselves and the already existing therapies all seem to target the same signaling pathway underlines the importance of targeting this pathway when treating NETs.

## Figures and Tables

**Table 1:** Tissue used for immunohistochemistry, in situ hybridization and qPCR.

Patient	Age/Sex	Location	Ki67 Index	Treatment
1	65/F	Colon	1%	Surgery
2	69/F	Small intestine	3%	Surgery/SSA
3	62/M	Ileum	2%	Surgery
4	55/F	Small intestine	7–8%	Surgery
5	64/F	Ileocecal	1%	Surgery/SSA

**Table 2:** Human Somatostatin Receptor Subtype Specificity (IC50-nM) [30,31].

Compound		Somatostatin Receptor Subtype					Dopamine
		1	2	3	4	5	
Somatostatin 14		1.95	0.25	1.2	1.77	1.4	
Somatostatin 28		1.86	0.31	1.3	5.4	0.4	
BIM-23014 (Lanreotide)		>1000	0.75	98	>1000	12.7	
BIM-23023		>1000	0.42	87	2.7	4.2	
BIM-53097							22.1
BIM-23A760		622	0.03	160	>1000	42.0	15

**Table 3:** Primer sequences.

MiRNA	Mature Sequence	Product ID
miR-7	UGGAAGACUAGUGAUUUUGUUGU	000268
miR-148a	UCAGUGCACUACAGAACUUUGU	000470
MiR-191	CAACGGAAUCCCAAAAGCAGCUG	002299
RNU-44	CCTGGATGATGATAGCAAATGCTG-ACTGAACATGAAGGTCTTAATTAGCTCTAACTGACT	001094

**Table 4:** Up- and down-regulated miRNAs based on Somatostatin analogue (SSA) treatment.

miRNA	Change	BIM-23014		BIM-23A760		BIM-53097	
		FC	*p*-Value	FC	*p*-Value	FC	*p*-Value
miR-769-3p		−2.8	0.001	−2.6	0.002	−1.1	NS
miR-663b		−2.0	0.009	−2.5	0.001	−2.9	0.00
miR-663	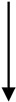	−2.0	0.007	−2.3	0.002	−3.3	0.00
miR-30b-star		−2.0	0.001	−2.2	0.000	−1.3	NS
miR-297		−1.7	0.008	−2.0	0.001	−1.7	0.01
miR-483-5p		−1.9	0.003	−2.0	0.002	−1.0	NS
miR-376c		2.1	0.000	2.0	0.000	−1.2	NS
Let-7f		1.8	0.001	2.1	0.000	−1.2	NS
miR-10a-star	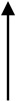	1.9	0.001	2.1	0.000	1.1	NS
miR-495		2.0	0.001	2.2	0.000	1.6	0.01
**miR-7**		2.3	0.000	2.2	0.000	−1.1	NS
miR-9-star		2.3	0.000	2.3	0.000	−1.4	NS
miR-454		1.9	0.001	2.3	0.000	−1.0	NS
miR-26b		2.2	0.010	2.6	0.003	1.0	NS
miR-429		2.9	0.004	2.8	0.004	−1.4	NS
miR-9		1.5	NS	2.9	0.002	−1.5	NS
**miR-148a**		2.5	0.006	2.9	0.002	2.0	0.03
miR-30e-star		2.8	0.000	3.1	0.000	1.5	NS

The up and down regulated miRNAs in NIH-H727 cells after 24 h treatment with somatostatin analogue BIM-23014, dopamine analogue BIM-53097 and the chimeric somatostatin-dopamine analogue BIM-23a760. Cut-off *p* ≤ 0.001, N = 4. NS—*p*-value not statistically significant; Fold Change—FC.

**Table 5:** Differentially regulated let-7 family members based on SSA treatment.

miRNA	Change	BIM-23014		BIM-23A760		BIM-53097	
		FC	*p*-Value	FC	*p*-Value	FC	*p*-Value
**let-7a**	→	1.2	0.000	1.2	0.000	−1.0	NS
**let-7b**	↓	−1.4	0.000	−1.4	0.001	−1.0	NS
**let-7c**	→	−1.0	NS	1.0	NS	−1.1	NS
**let-7d**	→	−1.0	NS	1.0	NS	1.1	NS
**let-7e**	→	−1.2	NS	−1.1	NS	1.1	NS
**let-7f**	↑	1.8	0.002	2.1	0.000	−1.2	NS
**let-7g**	↑	1.7	0.001	1.9	0.000	−1.1	NS
**let-7i**	↑	1.3	0.000	1.4	0.000	−1.1	NS
**miR-98**	↑	1.8	0.004	2.2	0.000	−1.1	NS

The alteration in the expression of the let-7 family of miRNAs in NIH-H727 cells after 24 h treatment with somatostatin analogue BIM-23014, dopamine analogue BIM-53097 and the chimeric somatostatin-dopamine analogue BIM-23a760. N = 4, NS—*p*-value not significant.
